# Site-control of InAs quantum dots on InP by droplet epitaxy in MOVPE

**DOI:** 10.1038/s44310-026-00139-z

**Published:** 2026-06-04

**Authors:** Guoliang Zhou, Elisa M. Sala, Young In Na, Paul W. Fry, Dominic J. Hallett, Alistair J. Brash, Luke Wilson, Jon Heffernan

**Affiliations:** 1 Department of Electronic and Electrical Engineering, The University of Sheffield, North Campus, Sheffield, UK; 2 EPSRC National Epitaxy Facility, The University of Sheffield, North Campus, Sheffield, UK; 3 School of Mathematical and Physical Sciences, The University of Sheffield, Sheffield, UK

**Keywords:** Materials science, Nanoscience and technology, Optics and photonics, Physics

## Abstract

We demonstrate a novel approach for site-controlled growth of InAs quantum dots on InP substrates emitting at telecom wavelengths, including the C-band, for quantum photonics applications. Regular nanohole arrays were defined by electron-beam lithography and dry etching, followed by InP regrowth. Quantum dots were then formed via droplet epitaxy in MOVPE, in which indium droplets were localised within the nanoholes and subsequently crystallised into site-controlled InAs quantum dots. Micro-photoluminescence measurements on a quantum dot array (2.5 µm pitch over 14 × 14 sites) reveal single-line emission in the telecom bands, with narrow linewidths reaching the instrument resolution, approximately 50 µeV for 60% of the dots and a single dot occupancy for over 80% of the nanoholes in the array.

## Introduction

Self-assembled semiconductor quantum dots (QDs) have attracted considerable attention owing to their unique physical properties and their enormous potential in quantum technologies^1,2^. Their delta-function-like density of states enables on-demand emission of single and entangled photons, making them promising single-photon sources for applications such as quantum key distribution and qubits in quantum computing^3,4^. InAs/GaAs QDs exhibit outstanding single-photon emission properties and have been extensively developed as near-ideal photon source^5,6^. However, this material system is intrinsically limited to emission around 900 nm due to strain constraints, a wavelength that is not compatible with existing fibre-optic infrastructure because of its relatively high transmission loss (>1 dB/km)^7^. There are two approaches to achieve redshifted emission wavelengths. The first is to introduce a metamorphic buffer layer to modify the strain and affect the size of the InAs QDs^8,9^. However, this method requires the use of additional graded layers, which degrades the crystalline quality and increases the difficulty of device scalability^10^. The second approach is to switch the substrate to InP. InAs/InP QDs can naturally extend the emission wavelength to 1.55 µm, corresponding to the telecom C-band where fibre attenuation reaches its minimum (0.15 dB/km)^11^. We previously demonstrated the MOVPE growth of high-quality C-band InAs/InP QDs by DE on planar InP. More recently, we demonstrated Stark tuning and charge state control of single QDs^12^, as well as improved spin properties compared to their SK counterparts^13^. Therefore, InAs/InP QDs are well suited for enabling long-distance quantum communication and for integration with silicon-based photonic chips in quantum computing^14,15^. However, it is well known that self-assembled QDs grown by conventional methods are randomly and non-uniformly distributed, which severely hinders their scalable integration into devices^16^. Therefore, the capability to precisely control the positions of high-quality InAs/InP QDs is of critical importance^17^.

The Stranski-Krastonov (S-K) growth mode is a conventional method for QD growth and remains the most widely used technique^18^. However, the shape of QDs formed by strain is difficult to control^19^, and in the InAs/InP material system they are particularly prone to elongation due to the smaller lattice mismatch compared to GaAs, often leading to the formation of quantum dashes^20^. In comparison, droplet epitaxy (DE) first relies on the spontaneous formation of group-III metallic droplets, followed by the supply of group-V elements to crystallise the droplets into QDs. DE offers a broader range of material choices and enables strain-free initiation of growth. Notably, owing to the dominance of surface tension during droplet formation, DE-grown QDs tend to be more compositionally uniform and symmetric as also shown in our previous works^21,22^, leading to a fine-structure splitting (FSS) up to four times smaller than that of SK-grown QDs^23^. Although trion emission is suitable for single-photon sources and spin-photon entanglement protocols, where FSS is not a strict requirement^24,25^, low-FSS neutral exciton emission is essential for applications involving polarisation-entangled photon pair generation^26^. DE was originally discovered and developed in MBE and has only recently been applied to commercial MOVPE^27,28^.

To precisely control the spatial position of QD, namely site-control of QDs, the pre-patterning of substrates has been proposed and developed^29–31^. This involves creating nucleation sites on the substrate acting as seeds for the QD growth. However, site-controlled QDs often suffer from poor optical quality because the patterning process can introduce impurities and defects^32^. Regrowth of a buffer layer on the treated nanohole surface to separate the QDs from the fabrication interface is an effective approach to address this issue^33^. In addition, employing the strain-free nature of DE for site-control can circumvent the poor controllability of local strain inherent to SK growth^34^. Nevertheless, the potential of DE for realising site-controlled InAs/InP QDs has not yet been explored, motivating the present study.

**Fig. 1:**
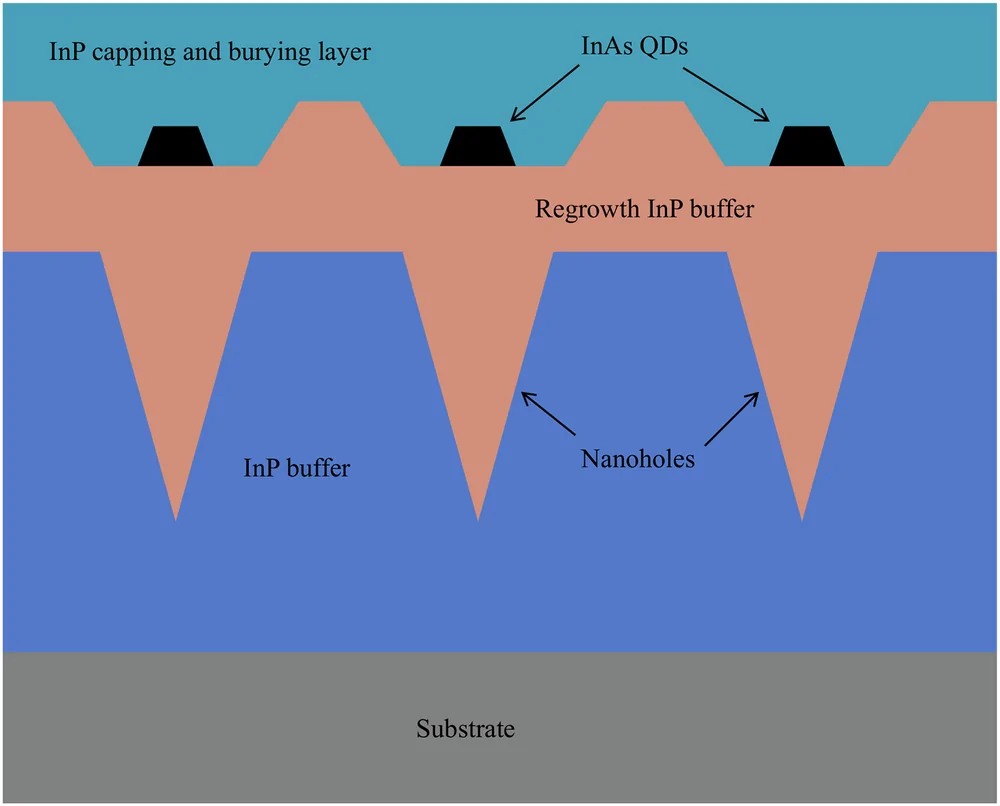
Design. The schematic diagram of fabrication and epitaxial structure.

In this work, we demonstrate site-controlled InAs QDs on InP substrates grown by DE using MOVPE, achieving single-line emission in the telecom C-band. DE-based site-controlled InAs QDs were fabricated on InP substrates by defining nanohole arrays using electron-beam lithography (EBL), followed by InP buffer growth, site-controlled formation of indium droplets, then their crystallisation into InAs QDs, and subsequent InP capping and burying layers regrowth. The structural schematic of the sample is presented in Fig. 1. Atomic force microscopy (AFM) characterisation at each stage confirmed the surface morphology and enabled observation of droplet and QD property during the DE process, while micro-photoluminescence (µPL) imaging and spectroscopy revealed the high optical quality of the resulting QDs.

## Results

### InP etching and buffer regrowth

**Fig. 2:**
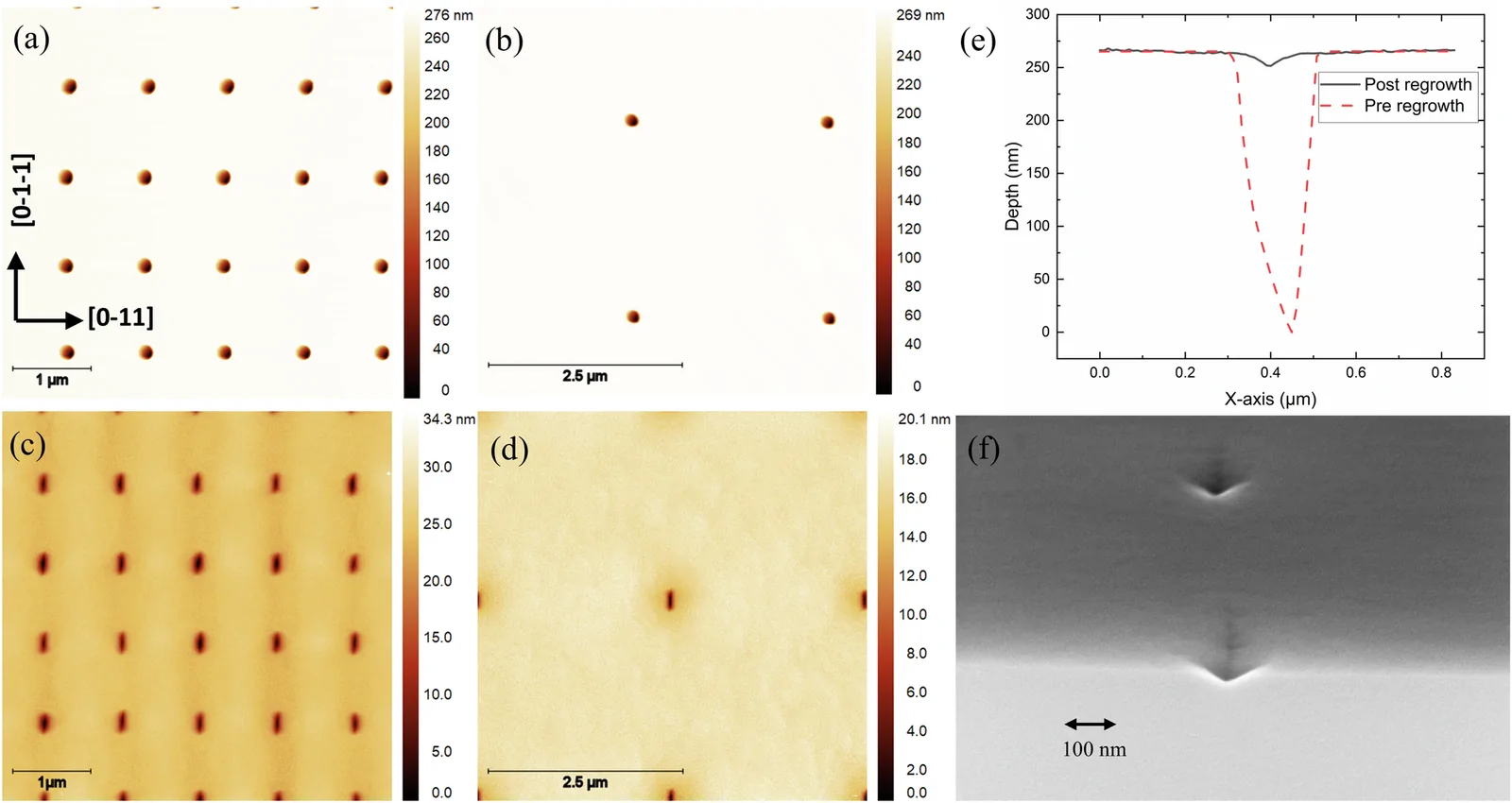
Morphological characterisation of nanoholes. AFM images of InP nanoholes after etching: **a** 1 µm pitch and **b** 2.5 µm pitch. AFM images of InP buffer regrowth (7 nm) on patterned substrates: **c** 1 µm pitch and **d** 2.5 µm pitch. All images were taken over a 5 µm × 5 µm scan area. **e** Pre and post regrowth profiles along the [0–11] crystallography direction. **f** Cross-sectional SEM image of regrown buffer layer structure.

AFM images of the nanohole arrays are shown in Fig. 2a, b for the 1 µm and 2.5 µm pitches, respectively. The e-beam dose was optimised to ensure that both patterns exhibited comparable depth and diameter. The nanoholes showed an average depth of 260 nm, with lateral dimensions of 211 nm along the [0-11] direction and 209 nm along the [0-1-1] direction. Overall, the fabricated nanoholes are deep and uniformly etched across the wafer.

A series of experiments was carried out where variable InP buffer thicknesses were grown on the nanohole patterns, ranging from a nominal 3 nm to 10 nm. A nonlinear growth behaviour was observed, in which the filling rate inside the nanoholes was approximately 30 times higher than that on the planar InP surface. This phenomenon has also been reported in previous studies^35^ and was attributed to the negative curvature at the bottom of the holes, which acts as a driving force for adatom diffusion and promotes preferential nucleation of the InP buffer. For thinner buffer layers, the QDs exhibited insufficient optical quality to emit single spectral lines due to defect-induced nonradiative recombination^32^ above following fabrication. In contrast, excessively thick buffer layers planarized the surface by nanohole in-filling, leading to the loss of site-control capability. An optimised thickness of 7 nm was identified as the best compromise, enabling single-line emission while still preserving the nanohole shape and thus site-control of the QDs. Therefore, in this work we focus on the 7 nm buffer layer. AFM images of the InP buffer regrowth are shown in Fig. 2c, d, corresponding to the two different pitches. The nanoholes are observed to elongate along the [0-1-1] crystallographic direction, which can be attributed to anisotropic migration of indium adatoms favouring this direction^36^. The profile line of a nanohole in the 2.5 µm pitch before and after regrowth is presented in Fig. 2e. The nanohole depth was reduced from 263.8 nm to 15.58 nm, corresponding to a 35.46-fold difference between the growth rates inside and outside the nanoholes. It is worth noting that the etched nanohole profiles appear tilted, which differs from the vertical sidewalls typically expected from dry etching. This is likely due to AFM tip convolution artifacts resulting from the small lateral size and large depth of the nanoholes. To further examine the filling quality of the buffer layer, and to ensure no voids are present, a cross-sectional SEM image of a nanohole after buffer regrowth is shown in Fig. 2f. The bottom of the hole is found to be completely filled, with no evidence of void formation^37^, indicating that our growth conditions result in complete infilling.

### Site control of droplets and QDs

**Fig. 3:**
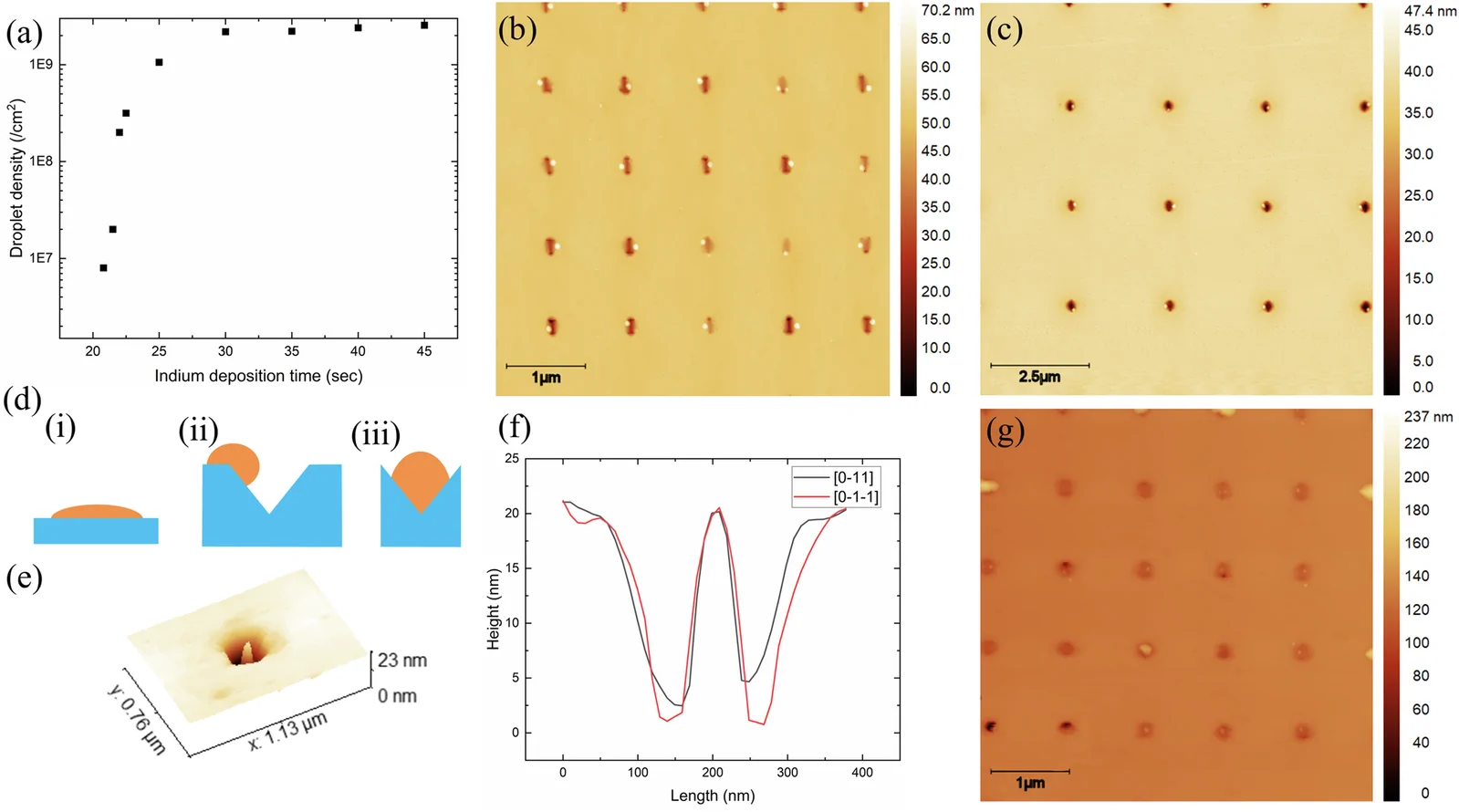
Morphology and properties of site-controlled droplets and QDs. **a** Indium droplet density as a function of indium deposition time. **b** AFM image of indium droplets on a 1 µm pitch nanohole array with a 25 s indium deposition time. **c** AFM image of indium droplets on a 2.5 µm pitch nanohole array with a 21.5 s indium deposition time, taken over a 10 µm × 10 µm scan area. **d** Schematic illustration of three possible droplet configurations on the surface: (i) on the planar surface, (ii) at the edge of the nanohole, and (iii) at the bottom of the nanohole. **e** 3D AFM image of a single nanohole containing one QD and **f** its corresponding profile line along the [0-11] and [0-1-1] crystallographic directions. **g** AFM image of InAs QDs on a 1 µm pitch nanohole array.

We subsequently conducted DE on this pre-patterned surface. The DE method allows independent control of droplet nucleation and crystallisation. Hence, our initial step was to localise the indium droplets. The key requirement was to ensure that the droplet density matched the density of the nanoholes, so that droplets would form exclusively inside the nanoholes, with exactly one droplet per hole in the ideal case. Our previous results on the dependence of indium droplet density on deposition time for planar InP are shown in Fig. 3a^38^. The droplet density increases sharply between 20.8 s and 25 s, then saturates approximately 30 s. When transferred to a patterned substrate, the density decreases by about 40%. Therefore, for the 1 µm pitch pattern, an indium deposition time of 25 s was selected. The resulting droplet density is 4.24 × 10^8^ cm^−2^, which is close to the nanohole density of 1.0 × 10^8^ cm^−2^. For the 2.5 µm pitch pattern, the indium deposition time was reduced to 21.5 s, based on Fig. 3a, to match the droplet density with the nanohole density. The growth results for the 1 µm and 2.5 µm pitch patterns are shown in Figs. 3b and 3c, respectively (the latter with a 10 µm scan size for improved statistical reliability). The droplets exhibit average heights of 15.45 nm and 13.22 nm, and widths of 72.16 nm and 67.12 nm for the 1 µm and 2.5 µm pitches, respectively. In both cases, droplets are exclusively localised within the nanoholes, with no droplets observed on the surrounding surface, corresponding to 100% occupancy and one droplet per nanohole. Therefore, site control of droplets was successfully achieved for nanohole arrays with different pitches by tuning the droplet deposition time.

It is noteworthy that the droplet positions were predominantly located near the edges of the nanoholes rather than at their centres. Three possible configurations of droplets on the surface are illustrated in Fig. 3d. Unlike the S–K growth mode, where QDs nucleate directly at the bottom of the holes because the local strain at the patterned sites reaches the critical value for dot formation earlier than elsewhere, as observed in the InAs/GaAs system^39^ and illustrated in Fig. 3d(iii), droplets formed via the DE process tend to nucleate at the hole edges, as shown in Fig. 3d(ii). This behaviour can be attributed to the migration of indium droplets driven by surface tension to minimise the overall surface energy. During this process, the droplets tend to remain pinned at the edge of the nanoholes, where the increased contact area compared to the planar case (Fig. 3d(i)) results in a lower surface energy^38^. The nanohole sidewalls, which are essentially planar surfaces, act as potential barriers that prevent further migration of the droplets toward the bottom of the holes illustrated by the calculated chemical potential map of the nanohole surface in this study^40^, where a local minimum in chemical potential appears at the edge of the nanohole.

After the droplet localisation was completed, the droplets were crystallised into InAs QDs by exposure to an Arsenic overpressure^41,42^. Figure 3g shows the site-controlled QDs for 1 µm pitch. A 100% occupancy was achieved, with exactly one QD per nanohole, confirming that the position established during droplet localisation is maintained after crystallisation. A representative 3D morphology (Fig. 3e) and the corresponding profile (Fig. 3f) are also presented. The QD exhibits a height of 15.42 nm, with widths of 83 nm along the [0-11] direction and 88 nm along the [0-1-1] direction. This result is highly promising, particularly as the QD profile exhibits a symmetric shape, which can be attributed to the unique advantages of DE. The symmetry of the site-controlled QDs observed here is consistent with that previously reported for DE-grown InAs/InP QDs on unpatterned substrates^41^. It is noteworthy that during crystallisation, the QDs migrated to the bottom of the nanoholes. This phenomenon can be understood using the Srolovitz model^43^, which describes the stability of a strained solid surface; the surface chemical potential is expressed as^44^

1 $$\mu \left(x\right)={\mu }_{0}+\gamma \varOmega \kappa \left(x\right)+\varOmega {E}_{s},$$

where ${\mu }_{0}$ is the chemical potential of flat surface, γ is the surface free energy, Ω is the atomic volume, κ(x) is the local surface curvature, and ${E}_{s}$ is the strain energy density. Adatoms follow the mechanism of diffusing from regions of high chemical potential to regions of low chemical potential. According to Eq. (1), the bottom of the nanohole exhibits the lowest chemical potential due to its negative surface curvature^35,40^, leading to the nucleation of QDs at the bottom of the holes. This is consistent with the experimental observations, as shown in Fig. 3e. The tendency of QDs to localise at the bottom of the nanoholes is advantageous for achieving precise site control, as it allows their positions to be accurately defined. However, the full dynamics of the crystallisation process remain unclear. As discussed in previous work^21^, indium outdiffusion and arsenic exchange during droplet crystallisation may also play significant roles in determining the final QD position.

It can also be observed that, along with the formation of QDs, some large defects are generated. This is attributed to the redistribution of material during the cooling process after crystallisation, whereby excess material can migrate into the nanoholes. A greater number of such large defects are found for the 2.5 µm pitch, since fewer nanoholes are available to accommodate the excess material (not shown). However, after the growth of a 100 nm InP capping layer, the surface becomes essentially planarized and these large features, which can reach several hundred nanometres in height, disappear. This suggests that the capping layer effectively prevents further material from entering the nanoholes, and that the large defects are a function of the cooling of uncapped dots only.

### Optical characterisation of site-controlled QDs

**Fig. 4:**
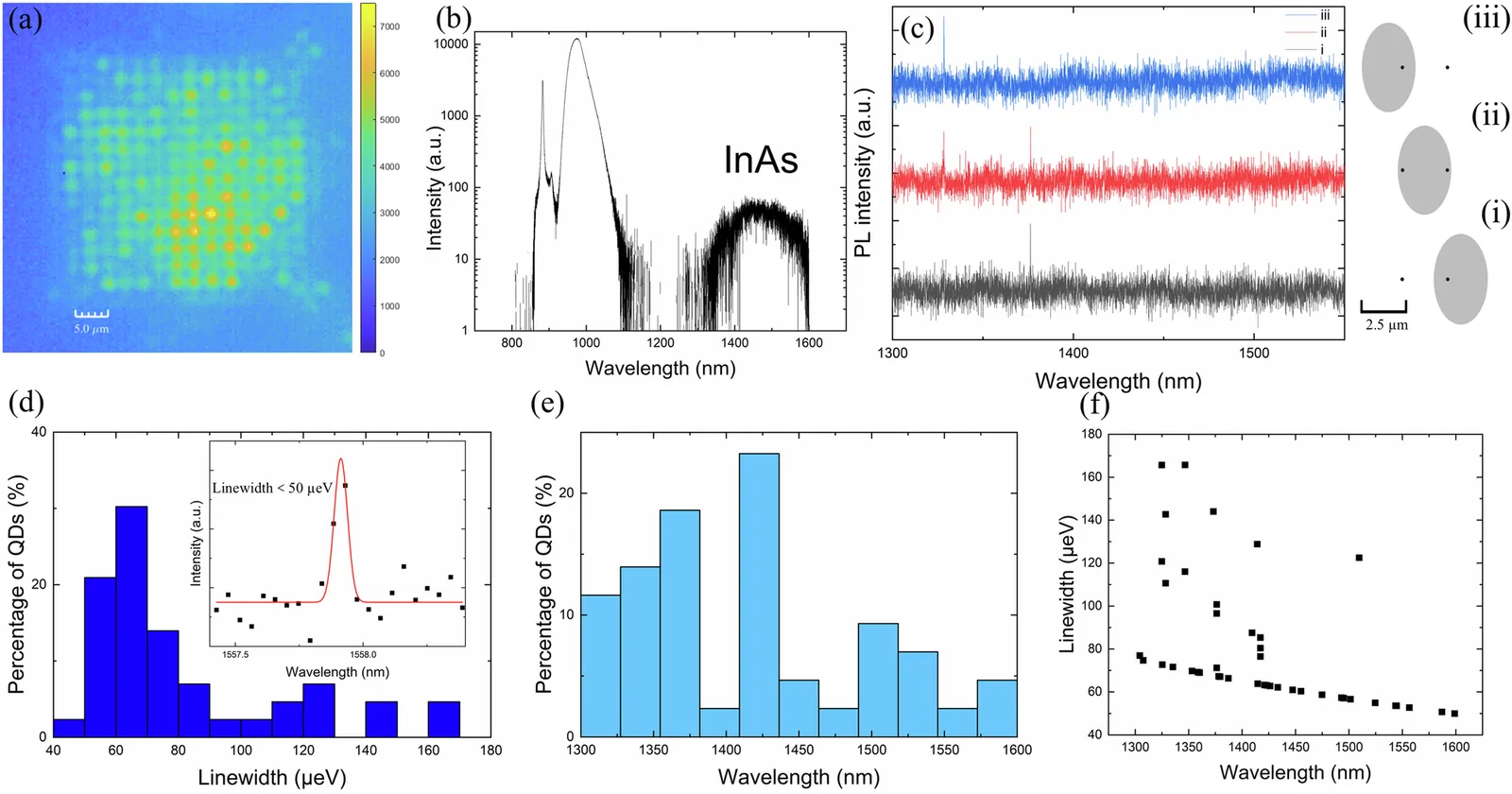
Spatially resolved μPL imaging and spectroscopy of site-controlled QDs. **a** µPL image of the site-controlled QD area at 4 K. **b** Low-temperature (*T* = 4 K) high-power µPL measurement of the same site-controlled QD area. **c** Sharp emission lines in low-power µPL mapping reveal site-dependent behaviour. The schematic next to the spectra illustrates the relative positions of the laser spot (3 µm wide ellipse) and two QDs (2.5 µm pitch). **d** Linewidth distribution of single dot emissions. The Inset presents a high-resolution 1 nm spectral window of a single QD emission at the spectrometer resolution limit, with a linewidth below 50 µeV at 1558 nm. **e** Wavelength distribution of single dot emissions. **f** Linewidth as a function of emission wavelength for individual QDs.

Having established the physical site control of QDs, we then investigated the optical properties of the QDs by µPL measurements. The optical results in this section were all obtained from the 2.5 µm pitch samples, as the spacing is sufficient to ensure spatial isolation. Figure 4a shows the µPL image of the patterned area, measured using an InGaAs camera fitted with a 1300 nm long-pass filter to suppress the shorter-wavelength background emission. The measurement was performed under non-resonant excitation using an 810 nm LED source. An LCPLAN N 50× IR objective lens was used together with a 200 mm tube lens (TTL200-S8) to focus onto the camera, resulting in a field of view of approximately 200 µm. Each camera pixel corresponds to ~250 nm, and the imaging resolution at 1550 nm is ~1.2 µm. The power density in our measurement was 33.74 W/cm². The resolution is sufficiently high to clearly resolve individual QDs within the array. In a 14 × 14 QD array, four cross-shaped alignment markers can be seen at the corners, and 83% of sites exhibit emission, corresponding to a high QDs occupancy. Notably, one possible reason for the absence of emission from the remaining sites is that some QDs may emit beyond the spectral cut-off of the camera at 1700 nm. Power-dependent measurements on emissive sites showed a power-law dependence and characteristic saturation behaviour at high excitation levels, consistent with excitonic emission from single QDs^45^.

Figure 4b presents spectrally resolved µPL measurement performed at 4 K under high-power non-resonant excitation at 635 nm. A broad emission band ranging from 1300 nm to 1600 nm is attributed to the ensemble emission of QDs. In addition, two other peaks are observed: one around 880 nm originating from the InP substrate^46^, and another around 980 nm attributed to the quasi-wetting layer^47^. Although the DE process itself does not produce a wetting layer, a thin quasi-wetting layer of InAs_*x*_P_1−*x*_ can form during crystallisation due to As–P exchange, as previously investigated^21^.

After the capping process, it was not clear whether the one dot per nanohole correspondence observed in the uncapped samples by AFM was preserved. To verify this and quantitatively assess the optical quality of the QDs, additional µPL mapping was performed on the patterned area under low excitation power at 4 K. Figure 4c presents spatial scans that confirm one QD per site. This figure illustrates the relative positions of the laser spot and the QDs. The excitation laser spot, with an elliptical size of 3 µm × 5 µm, directly excites a single QD but does not reach the neighbouring dot 2.5 µm away, resulting in only one emission line, as shown in Fig. 4c-i. When the laser spot is shifted to the left, another peak appears, indicating that the excitation area now covers an additional QD (Fig. 4c-ii). As expected, with a further shift to the left, the first peak disappears while the second peak remains (Fig. 4c-iii), providing direct evidence that the QDs are site-controlled with exactly one QD per nanohole^48^.

Figure 4d presents the distribution of emission linewidths. Statistical analysis yields an average linewidth of 82 µeV, but 60% of the single lines reaching our spectrometer resolution limit. Compared to self-assembled QDs, the site-controlled QDs exhibit a significantly larger fraction of resolution-limited emission lines. We attribute this to the introduction of nanoholes, which provide geometric confinement, leading to improved control over QD size and morphology. Thus, the overall distribution of linewidths in the site-controlled sample is reduced, and with the growth optimisation we have performed, that distribution is weighted towards the spectrometer resolution. The reduced size dispersion suppresses inhomogeneous broadening and likely mitigates spectral diffusion, resulting in spectrally narrower emission lines. The inset of Fig. 4d shows a high-resolution 1 nm spectral window of a single QD emission that reaches the spectrometer resolution limit, exhibiting a linewidth below 50 μeV at 1558 nm, without deconvoluting the instrumental response. This emission originates from a site-controlled QD operating in the telecom C-band, suitable for quantum photonics applications. Combining these results, we conclude that the site-controlled InAs/InP QDs fabricated in this work exhibit high optical activity and narrow linewidths.

The wavelength distribution of these single emission lines is analysed in Fig. 4e. The emission spans the entire telecom window, including the C-band, consistent with the broad spectral range from 1300 to 1600 nm shown in Fig. 4b. Figure 4f displays the linewidth as a function of emission wavelength. Linewidths approaching the resolution limit are observed across the full spectral range. Notably, broader linewidths are primarily concentrated in the shorter-wavelength region (1300–1425 nm), while such broadening is absent at longer wavelengths. This behaviour may originate from increased size dispersion among the smaller QDs that emit at shorter wavelengths, resulting in enhanced inhomogeneous broadening^49^. The challenge of achieving narrower linewidth and wavelength distributions will be addressed in future work.

**Fig. 5:**
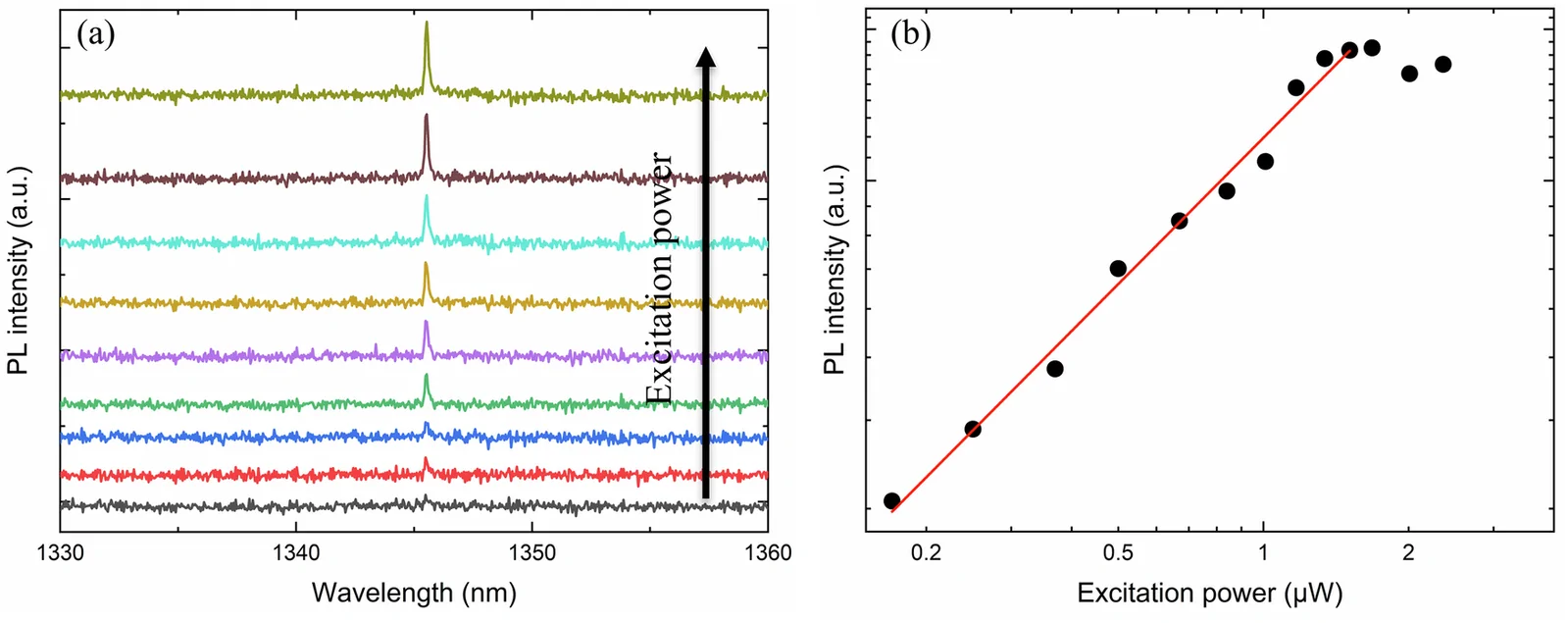
Excitation power-dependent measurements. **a** Power-dependent µPL spectra of a single quantum dot. **b** Integrated intensity of the single peak from **a** as a function of excitation power. A linear fit with a slope of 0.97 was employed for the X transition.

Finally, power-dependent measurements were performed on a representative single quantum dot. Figure 5a shows a series of µPL spectra under increasing excitation power, where only a single emission peak is observed. The corresponding integrated intensity is plotted as a function of excitation power in Fig. 5b, yielding a linear fit with a slope of 0.97. At higher excitation powers, the emission saturates. This behaviour confirms that the peak originates from the neutral exciton (X) transition. No biexciton emission is observed from the single dot, and this observation is consistent with the power-dependent measurements performed using the µPL camera setup.

## Discussion

In summary, we have presented a method for realising site-controlled InAs/InP QDs grown by DE in MOVPE, achieving deterministic positioning and high optical quality emission in the telecom C-band. Site control was achieved through localisation of Indium droplets within nanoholes, which was retained after crystallisation to produce one QD per site. The fabricated QD arrays exhibited a high yield, with no dots between sites and good optical properties, with linewidths reaching the spectrometer resolution limit in over 60% of the measured QDs within a 14 × 14 array. These results demonstrate the potential of DE-based site-controlled InAs/InP QDs as scalable arrays of single or entangled photon emitters for integration into quantum photonic circuits operating in the C-band.

## Methods

### Nanohole fabrication

First, a 0.5-µm InP buffer layer was grown by MOVPE on commercial InP (100) substrates to improve the surface morphology prior to fabrication, achieving a root-mean-square (RMS) roughness as low as 0.218 nm. Subsequently, a 100-nm-thick SiN layer was deposited by plasma-enhanced chemical vapour deposition (PECVD) as a hard mask. EBL was then performed on a spin-coated high-resolution positive tone resist (CSAR-62), followed by development to create nanoholes of 100 nm diameter. The patterned arrays consisted of nanoholes with a range of pitches, among which the 1 µm and 2.5 µm pitch arrays are discussed in detail. Reactive ion etching (RIE) and inductively coupled plasma (ICP) etching were sequentially used to etch the SiN and InP layers, respectively. Finally, the remaining SiN was removed using hydrofluoric acid (HF), resulting in nanoholes with diameters of ~200 nm and depths of ~250 nm. The samples were then loaded into the MOVPE reactor for subsequent epitaxial growth.

### Epitaxial growth

All samples were grown in a close-coupled showerhead (CCS) MOVPE reactor using trimethylindium (TMIn), phosphine (PH₃), and arsine (AsH₃) as precursors and hydrogen (H₂) as carrier gas. The growth started with the nanohole array regrowth of an InP buffer layer in order to keep subsequent QD growth away from the fabrication interface and minimise surface defects. The 250 nm deep nanoholes were infilled to a final depth of 15 nm with an InP buffer grown at 500 °C at a growth rate of 0.5 µm/h and a V/III ratio of ~500. The temperature was then lowered to 320 °C for indium droplet formation, followed by crystallisation into QDs under an AsH_3_ flow while the temperature ramped to 520 °C. The detailed procedures have been outlined in our previous works^41,42^. For AFM characterisation of free-standing surface QDs, the sample is immediately cooled down and taken out of the growth chamber, while for samples intended for µPL measurements, a 100 nm thick InP burying layer was grown using a double-cap growth procedure^50^.

## Data Availability

The datasets generated and analysed during the current study are available in the University of Sheffield Online Research Data (ORDA) repository. DOI: 10.15131/shef.data.31584121.
